# Accelerated Weathering Effects on Poly(3-hydroxybutyrate-*co*-3-hydroxyvalerate) (PHBV) and PHBV/TiO_2_ Nanocomposites

**DOI:** 10.3390/polym12081743

**Published:** 2020-08-05

**Authors:** Ana Antunes, Anton Popelka, Omar Aljarod, Mohammad K. Hassan, Peter Kasak, Adriaan S. Luyt

**Affiliations:** Center for Advanced Materials, Qatar University, P.O. Box 2713 Doha, Qatar; ana.antunes@qu.edu.qa (A.A.); anton.polelka@qu.edu.qa (A.P.); o.y.aljarod@qu.edu.qa (O.A.); mohamed.hassan@qu.edu.qa (M.K.H.); peter.kasak@qu.edu.qa (P.K.)

**Keywords:** poly(3-hydroxybutyrate-co-3-hydroxyvalerate), rutile titanium (IV) dioxide, accelerated weathering degradation, morphology and properties

## Abstract

The effect of accelerated weathering on poly(3-hydroxybutyrate-co-3-hydroxyvalerate) (PHBV) and PHBV-based nanocomposites with rutile titanium (IV) dioxide (PHBV/TiO_2_) was investigated. The accelerated weathering test applied consecutive steps of UV irradiation (at 340 nm and 0.76 W m^−2^ irradiance) and moisture at 50 °C following the ASTM D4329 standard for up to 2000 h of exposure time. The morphology, chemical structure, crystallization, as well as the mechanical and thermal properties were studied. Samples were characterized after 500, 1000, and 2000 h of exposure time. Different degradation mechanisms were proposed to occur during the weathering exposure and were confirmed based on the experimental data. The PHBV surface revealed cracks and increasing roughness with the increasing exposure time, whereas the PHBV/TiO_2_ nanocomposites showed surface changes only after 2000 h of accelerated weathering. The degradation of neat PHBV under moisture and UV exposure occurred preferentially in the amorphous phase. In contrast, the presence of TiO_2_ in the nanocomposites retarded this process, but the degradation would occur simultaneously in both the amorphous and crystalline segments of the polymer after long exposure times. The thermal stability, as well as the temperature and rate of crystallization, decreased in the absence of TiO_2_. TiO_2_ not only provided UV protection, but also restricted the physical mobility of the polymer chains, acting as a nucleating agent during the crystallization process. It also slowed down the decrease in mechanical properties. The mechanical properties were shown to gradually decrease for the PHBV/TiO_2_ nanocomposites, whereas a sharp drop was observed for the neat PHBV after an accelerated weathering exposure. Atomic force microscopy (AFM), using the amplitude modulation–frequency modulation (AM–FM) tool, also confirmed the mechanical changes in the surface area of the PHBV and PHBV/TiO_2_ samples after accelerated weathering exposure. The changes in the physical and chemical properties of PHBV/TiO_2_ confirm the barrier activity of TiO_2_ for weathering attack and its retardation of the degradation process.

## 1. Introduction

Petroleum-based polymers present substantial environmental problems, not only in the production process, but also at the end of life, because of their slow degradation and harmful degradation products. Biodegradable polymers have the advantage of a low environmental impact and high sustainability [1].

Poly(3-hydroxybutyrate-co-3-hydroxyvalerate) (PHBV), one of the most used polyhydroxyalkanoates (PHAs), is a linear, hydrophobic thermoplastic, and a semicrystalline polyester and biodegradable polymer. Its physical and chemical properties make it very attractive for several applications, such as a substitute for non-biodegradable polymers in medical uses such as antitumor and vascular system materials, as well as in pharmaceutical applications such as biocompatible drug delivery systems [2,3,4]. Moreover, PVBV-based materials have been used more and more in high performance food packaging, single-use and disposable items, housewares, electrical and electronics devices, agriculture and soil stabilization, adhesives, paints and coatings, and automotive parts [4,5,6].

On the other hand, polymer blending is an effective approach to boost some polymer characteristics, tailoring its physico–chemical properties and overcoming the specific limitations of the PHBV. Some of the polymers used for this purpose are poly(lactic acid) (PLA) [7,8,9,10], poly(ε-caprolactone) (PCL) [11,12], and poly(butylene adipate-co-terephtalate) (PBAT) [6,13]. The main focus is to improve their thermal stability, printability, flavor and odor barrier properties, as well as mechanical properties. Reinforcement with natural fibers (such as maple wood, bamboo or straw fibers) and nanofillers (such as graphene and derivatives, nanocellulose, nanoclays and nanometals) has also been a field of study for researchers searching for the previously cited properties, but also looking for the enhancement of the miscibility of the polymer blends [2].

The properties of PHBV depend strongly on the level and distribution of the 3-hydroxyvalerate (HV) units in the polymer chains. Increasing the co-monomer content or the side group length of the of HV units gradually disrupts the regular brittle structure of poly(3-hydroxybutyrate) (PHB), increases its toughness, and decreases its crystallinity, crystallization rate, glass transition, and melting temperature. Meanwhile, the biodegradability is enhanced when the HV content is higher [14].

Extensive research efforts have been focusing on the PHBV degradation characteristics and mechanisms when subjected to soil and microorganisms [11,15,16,17,18,19,20,21], ultraviolet (UV) radiation [22,23,24,25,26], hydrolytic [25,27,28,29,30,31,32] and thermal [16,33] conditions. The degradation of PHBV includes several competing mechanisms: the hydrolysis of the ester group, series of reactions initiated by free radicals, crosslinking reactions, and Norrish I and II mechanisms are the most described processes [22,34]. Due to the chain cleavage of the polymer during degradation, the molar mass, crystallinity and crystal structure, as well as the chemical/thermal/mechanical properties, are affected [11,15,16,17,18,19,20,21,22,23,24,25,26,27,28,29,30,31,32,33]. The degradation rates of PHBV under simultaneous conditions can be significant. When biodegradable polyesters are exposed to weathering, photo- and hydrolytic degradation take place. It is therefore important to study the simultaneous influence of UV and moisture on degradation processes under long-term weathering in order to better understand these products’ lifetime.

The photo- and hydrolytic degradation of biodegradable PHBV are well known. However, only a few studies have been conducted under a combination of photo- and hydrolytic degradation conditions during prolonged times of weathering exposure. Accelerated weathering chambers are used to delete this gap in degradation studies. Wei and McDonald [34] studied the effect of accelerated weathering exposure on PHBV films. Tests were conducted up to 1000 h in a xenon-arc weatherometer and the films were exposed to a repeated 2 h cycle of radiation (0.70 W m^−2^ at 340 nm at 70 °C) followed by 2 h of radiation plus water spray. Sadi et al. [22] investigated the photodegradation of poly(3-hydroxybutyrate) (PHB) using artificial UV radiation in an accelerated weathering QUV chamber. The weathering cycles were defined as 8 h under UV-A light (0.89 W m^−2^ at 340 nm) at 60 °C and 4 h in the dark under condensed water at 50 °C with 12 weeks of exposure time.

Fillers and additives were used to accelerate or retard the degradation process of the polymers for specific applications. The effect of starch [35], cellulose [36], glass fibers [37], graphene [38,39], nanoclays [40,41,42], ZnO [43,44,45] and TiO_2_ [44,46,47,48,49,50,51,52] on the biodegradation of biopolymers has been studied, particularly in poly(lactic acid), which is the most studied biopolymer because of its versatility and relatively cheap price [53]. Loading these materials can improve the biodegradability of the polymer matrix, enhance its performance and increase the range of applications. Poly(butylene succinate), polycaprolactone and PHBV nanocomposites have also gained interest because of their properties [24,25,26,32,43,54,55,56,57,58,59,60].

Titanium dioxide (TiO_2_) is among the most described fillers and is commonly utilized as a photocatalytic material [53]. TiO_2_ has different crystallographic phases, i.e., anatase, rutile, and brookite, which confer distinct characteristics to itself [50]. Some authors described TiO_2_ as a UV blocking additive [61] instead of a photo-catalyzer [50,62]. Rutile has a substantially lower photo-catalyzing activity than anatase [51,62]. It is more suitable for being used as a UV blocker, and for white pigments and coatings because of its great refractivity and notable chemical inertia [50,51,62]. Anatase exhibits a higher photo-catalytic activity and is more suitable for being a photo-catalyzer which accelerates the photodegradation of polymer matrices [50,51,62].

The hydrophilicity of TiO_2_ should also be considered during a degradation process in the presence of water. Hydrophilic fillers, such as fibers and clays, are reported to enhance the water transfer (sorption and diffusion) properties of PHBV [63]. Badia et al. [32] studied the influence of the percentage of sisal fiber in PHBV biocomposites in terms of water absorption capability and water diffusion rate. They found that both increased with the percentage of fiber. Others authors [64,65,66] observed the same trend after adding different clays to PLA, which accelerated the biodegradation of this biopolymer. Luo et al. [47] noted that the biodegradation of PLA under composting conditions could be controlled by adding TiO_2_ nanofillers. The main reason was the relative hydrophilicity of TiO_2_ nanoparticles which allows an easier penetration of water into the polymer matrix, activating the hydrolytic degradation process [67].

However, the degradation rate of biopolymers can also be retarded using nanofillers because of their barrier effect when loaded into a polymer matrix. It was confirmed by Someya et al. [68] and Wu et al. [69] after producing nanocomposites of PLA containing montmorillonite. Similarly, ZnO was shown to act as a barrier against the permeation of water vapor and other volatile compounds when blended with PHBV, inhibiting the gain or loss of moisture from the nanocomposites [70].

To the best of our knowledge, although the degradation of PHBV was extensively studied, the weathering of PHBV composites, reinforced with nano-sized particles of TiO_2_ under simultaneous photo and hydrolytic degradation for long time periods has not been reported. Only Buzarovska et al. [25] studied the presence of 0.5–10 wt% TiO_2_ (ca. 80% anatase and 20% rutile) in a PHBV matrix, and achieved a significant acceleration of the degradation rates. Photocatalytic decomposition of poly(butylene succinate) was found to be clearly dependent on the size and dispersibility of anatase TiO_2_ particles by Miyauchi et al. [54]. Larger fractions and better dispersibility of smaller size particles enhanced the degradation. However, a recent study reported that the photodegradation of poly(butylene succinate-*co*-butylene adipate) was not affected by the addition of rutile TiO_2_ nanoparticles coated with a silicon–aluminum composite [60]. On the other hand, Antunes et al. [71] demonstrated the effect of rutile TiO_2_ nanoparticles on the accelerated weathering degradation of poly(lactic acid). The presence of TiO_2_ nanoparticles not only improved the cold crystallization behavior of the PLA, acting as nucleating agents, but the nanoparticles also catalyzed the UV-initiated degradation of the PLA. Therefore, these results support the hypothesis that weathering degradation under moisture and the UV exposure of PHBV loaded with rutile TiO_2_ should be clarified.

The aim of the work reported in this paper was to investigate the degradation process of PHBV under controlled conditions of accelerated weathering, and to obtain a better understanding of the long-term degradation of PHBV, particularly PHBV with a high HV content of 25%. We also investigated PHBV-based composites with 3 wt.% TiO_2_ under the same weathering conditions, to study the effect of TiO_2_ on the PBHV degradation. It was expected that the TiO_2_ nanoparticles would promote the weathering degradation of PHBV, as well as the biodegradability of PHBV containing a high level of HV. The accelerated weathering degradation test was performed up to a period of 2000 h.

## 2. Experimental

### 2.1. Materials

The PHBV containing 25% of 3-hydroxyvalerate segments in the polymer was obtained from Pensieve Technology Co., Ltd. (Wuhan, China). It was light yellow with a density of 1.24 g cm^−3^, a melt flow index of 3.2 g/10 min, glass transition around −45 °C and a melting temperature of ~90 °C. The molar mass dispersity of the neat PHBV was 1.41, showing 59,228 and 83,362 g mol^−1^, respectively, for M_n_ and M_w_.

The rutile TiO_2_ nanopowder, with particle sizes < 100 nm and 99.5% purity, was also obtained from Pensieve Technology Co., Ltd.

Ethylene glycol (>98%, FLUKA, Morris Plains, New Jersey, USA), formamide (>98%, FLUKA) and ultra-pure water (prepared by Purification System Direct Q3, Millipore Corporation, Molsheim, France) were used as the testing liquids for wettability analyses.

### 2.2. Preparation Method

The polymer was first dried in an oven at 50 °C overnight before sample preparation. The nanocomposite with 3 wt.% of TiO_2_ was prepared by melt-mixing the rutile TiO_2_ nanopowder with the polymer using a Plastograph EC (Brabender GmbH, Duisburg, Germany) at 120 °C and 30 rpm screw speed for ten minutes. The neat PHBV and the PHBV/TiO_2_ nanocomposite were then placed between two pieces of stainless steel plate covered by a release foil, and molded into 1 mm thick sheets at the same temperature for 5 min using a hydraulic press (Carver, Inc., Wabash, IL, USA) at a pressure of 50 bar. The sheets were allowed to cool at room temperature and then cut into dumbbell and rectangular shapes as further described.

### 2.3. Accelerated Weathering Test

The accelerated weathering of the PHBV and PHBV/TiO_2_ samples was conducted in an accelerated weathering tester Model QUV/se (Q-LAB, Westlake, Ohio, USA). The weathering conditions were following the Cycle-C of the ASTM D4329 standard. Fluorescent UV lamps (UV-A-340) with 0.76 W m^−2^ irradiance (wavelength 340 nm) were used with cycles of 8 h UV irradiation at 50 °C, followed by 4 h dark at 50 °C under 100% condensing humidity. These consecutive cycles were applied to the specimens attached to the test panels without any interruption. The effects of the accelerated weathering were investigated for four exposure periods: 0, 500, 1000, and 2000 h. Samples were collected for analysis after each period, and they were designated as ‘PHBV/*x*h’, where *x* denotes the accelerated weathering period in hours. Neat PHBV was designated as PHBV/0 h.

### 2.4. Characterization Techniques

#### 2.4.1. Surface Visual Changes

After each accelerated weathering period, the visual inspection of the variations in color, gloss, transparency, and roughness of the specimens was carried out. The visual inspection was made by comparing photographic images of the unweathered and weathered samples.

#### 2.4.2. X-ray Diffraction Analyses (XRD)

XRD analyses were performed using an Empyrean (Panalytical, Almelo, The Netherlands) equipped with a Cu K_α_ (λ = 0.1540 nm) source. The generator was operated at 40 mA and 45 kV. Samples were scanned from 5° to 80° at a scanning rate of 0.0130° 2θ.

#### 2.4.3. Atomic Force Microscopy (AFM)

Detailed information about the three-dimensional changes in the surface topography of the PHBV surface (neat and nanocomposite) was obtained using AFM. An MFP-3 D system (Oxford Instruments Asylum Research, Santa Barbara, California, USA) equipped with an AC160 TS cantilever (Al reflex-coated Veeco model, OLTESPA, Olympus, Tokyo, Japan) was used for the image scanning and determination of the mechanical properties by the application of an amplitude modulation–frequency modulation (AM–FM) technique. This mode represents an extension of the standard tapping mode, while the AFM cantilever with tip was excited simultaneously at both its fundamental resonant frequency and another eigenmode. The fundamental resonance allowed the observation of the topographical structures of the sample and tracking of the frequency. The amplitude shift of the other eigenmode was used for an investigation of the mechanical properties. The obtained frequency shift Δf was used for an estimation of the interaction stiffness $\Delta k^{FM}$ through Equation (1):

(1) $$\Delta k^{FM}\approx 2k_{c}\frac{\Delta f}{f_{c}}$$

where *k_c_* is the cantilever spring constant and *f_c_* is the frequency of the cantilever eigenmode. Young’s modulus of the samples was obtained using a general Hertz model describing the contact mechanics between the AFM tip and the sample. A standard with a known Young’s modulus (1.81 MPa) was first used for the cantilever calibration by an evaluation of its elasticity. This cantilever elasticity was then used to obtain absolute values of the stiffness and Young’s modulus of the PHBV and PHBV/TiO_2_ samples on the entire surface area.

#### 2.4.4. Scanning Electron Microscopy (SEM)

SEM analyses of the samples were performed to obtain 2D images of the surfaces using a NanoSEM 450 electron microscope (FEI Nova, Hillsboro, Oregon, USA), at an accelerating voltage of 2–5 kV. A thin Au layer of a few nanometers thick was sputter-coated onto the surfaces and cross-sections of the samples to obtain high-resolution images and to avoid the accumulation of electrons on the measured layer.

#### 2.4.5. Surface Energy

The changes in surface wettability of the PHBV and PHBV/TiO_2_ composites were evaluated by static contact angle (CA) measurements using the sessile drop method. A surface energy analysis system OCA35 (DataPhysics, Filderstadt, Germany) equipped with a CCD camera was used for this purpose. Water, formamide and ethylene glycol were used as testing liquids to evaluate the total surface free energy, as well as the polar and dispersive components, using the conventional Owens–Wendt–Rabel–Kaelble method. A droplet of approximately 3 μL from each testing liquid was placed on the air-facing samples. The CA was calculated after approximately 3 s to allow the thermodynamic equilibrium between the liquid and the sample interface to be reached. The reported value for each testing liquid corresponds to the mean of at least five measurements taken on different parts of the substrate surface. The obtained CA values were subsequently used for an evaluation of the total surface energy, as well as its polar and dispersive components.

#### 2.4.6. Fourier Transform Infrared (FTIR) Spectroscopy

FTIR spectroscopy with an attenuated total reflectance accessory was used to identify the chemical composition of the samples after weathering tests. An FTIR Frontier spectrometer (PerkinElmer, MA, USA) equipped with a ZnSe crystal was used for these analyses, capturing data from 1.66 μm penetration depth, using an average of 8 scans with a resolution of 4 cm^−1^. In order to quantify the extent of degradation, the carbonyl index, as derived from a ratio of the intensity heights of C=O/CH, was quantified before and after the accelerated weathering tests for the samples. In addition, a quantitative analysis of the ratio of the crystalline/amorphous phases was conducted. The absorbance ratios of the 1720/1388 and 1713/1388 cm^−1^ were used to quantify the crystalline carbonyl (C=O) content, while 1730/1388 and 1740/1388 cm^−1^ were used to calculate the amorphous C=O content of PHBV and its composites [31,32,72,73].

#### 2.4.7. Differential Scanning Calorimetry (DSC)

The DSC analyses were performed in a DSC8500 (PerkinElmer, Massachusetts, USA) differential scanning calorimeter. The PHBV samples (5–10 mg) were heated from 30 to 100 °C, respectively, at 10 °C min^−1^. The melting enthalpies of PHBV were determined from the DSC curves. The degree of crystallinity for the PHBV and its composites was calculated according to Equation (2):

(2) $$X_{c}\left(\%\right)=\frac{\Delta H_{m}\;}{W\times \Delta H_{m}^{{}^{\circ}}}\;\times 100$$

where Δ*H_m_* is the measured melting enthalpy, Δ*H°_m_* is the enthalpy of melting of the 100% crystalline polymer, with a value of 109.5 J g^−1^ for PHBV [74], and *W* is the polymer mass fraction in the analyzed sample (1.00 for neat PHBV, and 0.97 for PHBV/TiO_2_ because the mass fraction TiO_2_ was 0.03 in all the nanocomposite samples). The cooling scan was used to analyze Δ*H_c_*, the enthalpy of crystallization, and a second heating scan (curves are not shown here) was used to analyze the glass transition temperature, *T_g_*.

#### 2.4.8. Thermogravimetric Analysis (TGA)

A TGA4000 (PerkinElmer, Waltham, MA, USA) thermogravimetric analyzer (TGA) was used to analyze the thermal degradation behavior of the samples. The analyses were done from 30 to 600 °C at a heating rate of 10 °C min^−1^ under nitrogen flow (20 mL min^−1^). The sample masses were 10–15 mg.

#### 2.4.9. Melt Flow Index (MFI)

The melt flow index of the neat PHBV and PHBV/TiO_2_ was measured according to the ASTM D1238 standard using a Melt Flow Indexer LMI-4000 (Qualitest, Fort Lauderdale, FL, USA) at 100 °C under a load of 2.16 kg and a cut-off time of 10 s. For each measurement, 4 g of the material was loaded in the instrument.

#### 2.4.10. Tensile Testing

The tensile tests were performed by means of a Lloyd LR 50 k Plus (Lloyd Instruments Ltd., Fareham, UK) universal testing machine at a stretching speed of 10 mm min^−1^ (ASTM D638) at ambient temperature. The gauge length was 25 mm, and the sample (dumbbell shape) width and thickness were 3.25 and 1 mm, respectively.

## 3. Results and Discussion

### 3.1. Surface Morphology

**Figure 1:**
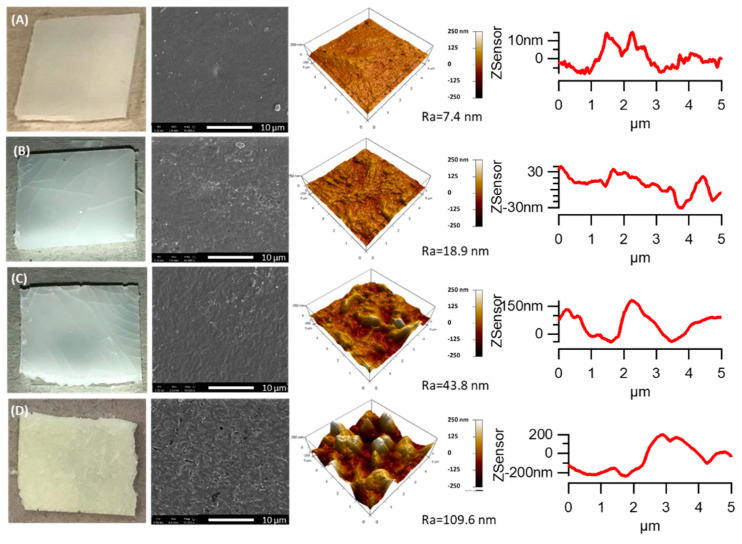
SEM and atomic force microscopy (AFM) images of the poly(3-hydroxybutyrate-co-3-hydroxyvalerate) (PHBV) samples before (**A**) and after accelerated weathering: (**B**) 500 h; (**C**) 1000 h; and (**D**) 2000 h. Note: R_a_ represents the roughness parameter.

The PHBV samples and PHBV/TiO_2_ nanocomposites were analyzed after 500, 1000, and 2000 h of accelerated weathering. Figure 1 shows the SEM and AFM images of the PHBV films over the course of accelerated weathering tests, indicating the susceptibility to degradation of the PHBV samples. Initially, the PHBV samples were ductile and presented smooth surfaces (Figure 1A). After 500 h (Figure 1B), the PHBV sample showed cracks formed on the surface. The formed cracks and crazing allowed the UV and moisture penetration into the bulk and accelerated the degradation process [34,75]. The cracks resulted from the contraction and expansion that occurred in the polymer during the drying (light) and wetting (light and condensation) cycles that enhanced the physical degradation, besides the chemical changes [34]. The physical degradation led to significant changes in the surface roughness when focusing on a small surface area (5 × 5 µm^2^), while the R_a_ value of the PHBV increased from 7.4 to 18.9 nm. After 1000 h of accelerating weathering exposure, a glossy surface was still observed, followed by a significant increase in the roughness of the surface (R_a_ = 43.8 nm). The sizes of the cracks increased with extended weathering (Figure 1C). For the samples after 2000 h of exposure, the PHBV turned slightly yellow and was completely degraded, and these samples reached the highest values of surface roughness (R_a_ = 109.6 nm) (Figure 1D).

**Figure 2:**
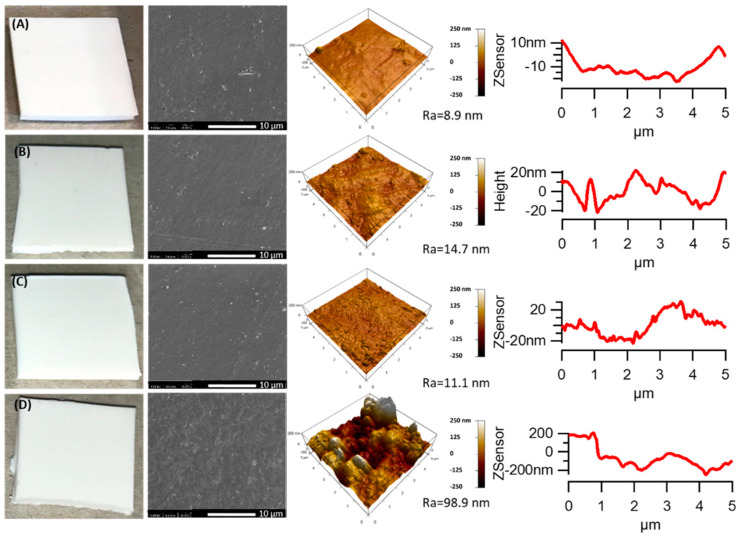
SEM and AFM images of the PHBV/TiO_2_ samples before (**A**) and after accelerated weathering: (**B**) 500 h; (**C**) 1000 h; and (**D**) 2000 h. Note: R_a_ represents the roughness parameter.

A different trend was observed in the PHBV/TiO_2_ samples, as is depicted in the SEM and AFM images of the PHBV/TiO_2_ over the exposed time (Figure 2). No cracks were visually detected for PHBV/TiO_2_, even after long periods of accelerated weathering (Figure 2, left photographs). SEM and AFM (Figure 2 middle and right images) also showed negligible changes in the analyzed surfaces after the accelerated weathering compared to the PHBV samples. This observation indicates the slower degradation of the PHBV/TiO_2_ nanocomposite compared to the PHBV sample. The PHBV/TiO_2_ surfaces continued to be uniform after 500 and 1000 h of accelerated weathering, without the formation of defects with negligible changes in roughness. However, the roughness increased significantly for the PHBV/TiO_2_ specimens weathered for 2000 h. The surface showed defects and contained cracks, and the R_a_ value changed from 8.9 nm (untreated) to 104.2 nm. It is possible to conclude that up to 1000 h, the degradation process was retarded through the action of TiO_2_, which could be useful for some applications when the degradation time needs to be controlled.

### 3.2. XRD Studies

**Figure 3:**
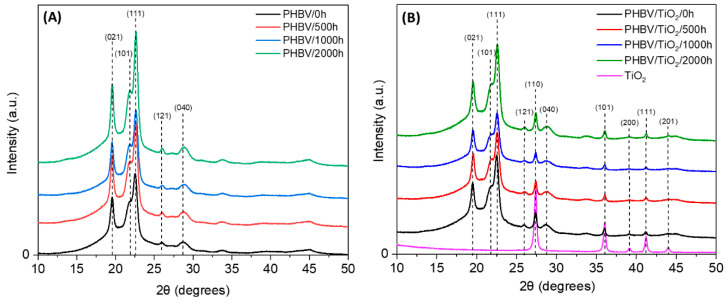
XRD spectra of (**A**) the neat PHBV and (**B**) the PHBV/TiO_2_ nanocomposites at different accelerated weathering times.

The effect of weathering exposure on the crystallinity of samples was first observed by XRD (Figure 3). Figure 3A shows the diffractograms of PHBV samples over the time of the accelerated weathering. The most intense reflection peaks of PHBV appeared at 19.6°, 21.7°, 22.5°, 25.9° and 28.7°, which correspond to the reflections of the (021), (101), (111), (121), (040) crystalline planes, respectively, and these peaks are in agreement with the previously observed PHBV structure [70,76]. However, the characteristic peaks at 13.6° and 17.1°, which correspond to the reflections of the (020) and (110) planes, were not observed. According to the literature, the morphologies of these crystals could be more or less dependent on processing variables such as thickness, temperature and pressure [77,78,79]. Different material morphologies such as powder, fibers and films [80,81], the HB/HV ratio [76] and the applied pre/post treatment [80,82] can also influence these peaks. No significant change was observed in the crystalline morphology of PHBV during the accelerated weathering test. However, the degree of crystallinity was calculated using the melting enthalpy, and it is presented in the DSC section.

Figure 3B depicts the XRD pattern of the PHBV/TiO_2_ nanocomposites over the exposure time, as well as that of the neat TiO_2_. The XRD profiles of the PHBV/TiO_2_ nanocomposites show the same peaks observed in the neat PHBV samples, as well as those from the TiO_2_ nanoparticles, observed at a 2θ of 27.4°, 36.1°, 39.2°, 41.2°, 44.0°, 54.2° and 56.6°, which correspond to the (110), (101), (200), (111), (201), (211) and (220) planes, respectively [83,84]. The XRD results from the nanocomposites suggest an overall increase in the crystallinity of the PHBV/TiO_2_ after 2000 h of accelerated weathering exposure, indicating that 2000 h of weathering exposure increased the crystallinity of this polymer [22,47,85]. It is further observable that the intensity of each peak is lower for the PHBV/TiO_2_ nanocomposites than for the neat PHBV sample, suggesting an extensive degradation of PHBV in the absence of TiO_2_, because of an observably higher crystallinity caused by the re-crystallization of the shorter chain segments after degradative chain scission (as was also observed in SEM analysis and further discussed in DSC). In line with the DSC results, the XRD results indicate lower crystallinities for the nanocomposite samples resulting from less degradation and chain scission.

### 3.3. Wettability Analyses

**Figure 4:**
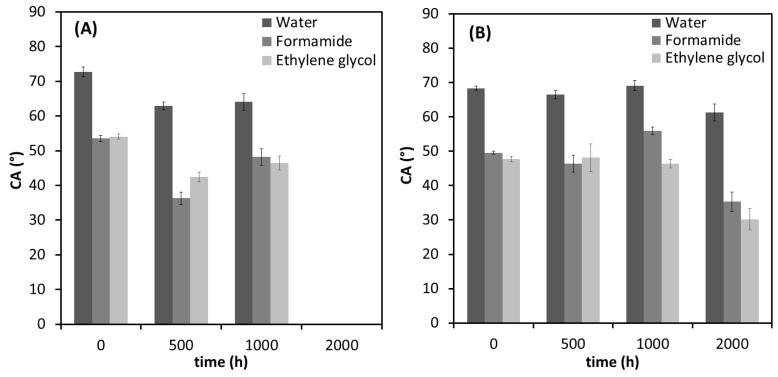
Contact angles (CAs) of water, formamide and ethylene glycol in (**A**) the PHBV and (**B**) the PHBV/TiO_2_ as a function of increasing weathering exposure time.

The hydrophilicity of polymeric materials plays an important role in their degradability characteristics. Figure 4 shows the CA results of water, formamide and ethylene glycol for the neat PHBV and the PHBV/TiO_2_ nanocomposites as a function of accelerated weathering time. The PHBV sample showed the highest value of CA for water, 72.7° ± 1.4°, and a decrease in the CA values was observed for PHBV/500 h and PHBV/1000 h (Figure 4A), which indicates that the hydrophobicity degree for the samples decreased. The first stage of PHBV degradation is usually the reduction of its molecular weight that occurs by the random cleavage of the ester bonds. As demonstrated by other authors [86,87], a longer carbon chain length enhances the hydrophobicity of the polyester, and therefore the lower CA values for water after 500 h of weathering confirms the degradative chain scission of the sample.

**Figure 5:**
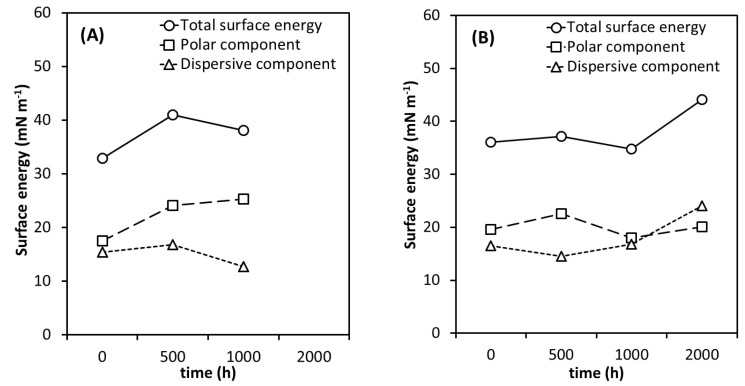
Surface energy of (**A**) the PHBV and (**B**) the PHBV/TiO_2_ as a function of increasing weathering exposure time.

Regarding the surface energy properties, an increase in the polar component over the weathering time can be observed in Figure 5A for the neat PHBV, while the surface energy and the dispersive component increased and then decreased. During polymer degradation, free radicals were generated reacting with ambient oxygen to produce peroxide groups. These compounds decompose and give rise to a variety of polar groups, such as hydroxyl, carbonyl, and carboxylic acid [22,32,34,37,88,89,90,91], which should be the reason for the observed continuous increase in the polar component and increase in CA for ethylene glycol and formamide after 1000 h. The FTIR results, that are discussed later on, corroborated with this trend. A gradual intensity decrease in all the spectra with increasing accelerated weathering time was observed for the neat PHBV, but the photo and hydrolysis degradation are strong evidence of the observed decrease in the amorphous phase of neat PHBV.

However, it does not explain the observed surface energy and the dispersive component profile over the exposure time. Besides the chemical composition of each material, the cracks and holes on the surface also contribute to the surface energy. The physical changes observed during degradation increase the roughness and this can also contribute to an increase in hydrophobicity [24]. Although the degradation observed in the AFM and SEM analyses continued with increasing exposure time, these results suggest a combination of chemical and physical degradation that contributed to the surface properties for neat PHBV. The modification of the PHBV/500 h surface was mainly the result of chemical alterations (number of polar groups as well as the carbonyl chain length), while physical properties take control between 500 and 1000 h of weathering. The CA of PHBV/2000 h could not be determined because its surface was completely degraded as observed in Figure 1, thus, it was not possible to correctly assess this value.

The CA of the water on the surface of the unweathered nanocomposites (Figure 4B) was lower than that in the case of neat PHBV, which means that the presence of TiO_2_ made the nanocomposites more hydrophilic than the neat PHBV. The higher hydrophilicity of the nanocomposites can be attributed to the hydrophilic character of the TiO_2_ nanoparticles [92]. All the PHBV/TiO_2_ samples presented similar CA values for water, ethylene glycol and formamide before and after weathering for up to 1000 h of exposure (Figure 4B), which was also observed in the surface energy (Figure 5B). It follows the same trend as was observed in SEM and the optical photographs. The presence of TiO_2_ in the PHBV/TiO_2_ nanocomposites indicate a retardation of the degradation processes compared to the PHBV samples. However, 2000 h of exposure to accelerated weathering resulted in a slight reduction of CA values for the three liquids, and an increase in surface energy of the PHBV/TiO_2_/2000 h sample. Chemical modifications of PHBV/TiO_2_/2000 h were also observed from the FTIR results—see the subsequent discussion. However, this degradation occurred simultaneously in the amorphous and crystalline phases, contrary to what was observed for the neat PHBV. The roughness recorded in the SEM and AFM for these samples, as well as the cleavage of the PHBV side-chains and degradative chain scission of both the amorphous and crystalline phases during the weathering, can be the reason for the observed decrease in the polar component, while the dispersive component increased. It is possible to conclude that the nanocomposites need a much longer time of weathering exposure to show changes in its surface energy.

### 3.4. Surface Chemistry (FTIR)

**Figure 6:**
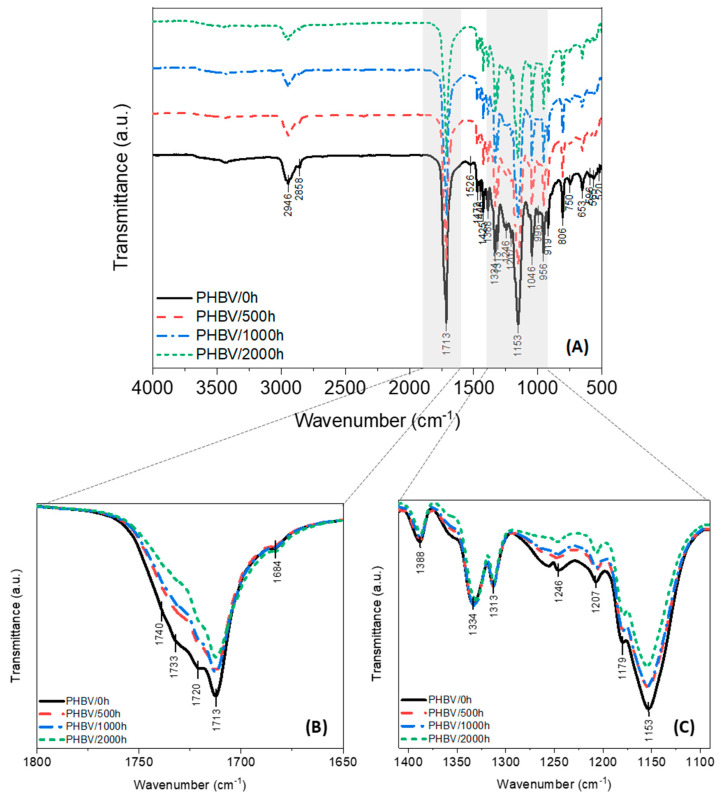
FTIR spectra of the neat PHBV before (0 h) and after (500, 1000 and 2000 h) accelerated weathering (**A**). Detailed area of: the stretching C=O vibration (**B**), the CH_3_ deformations (1338 cm^−1^), and the stretching C–O (1255–1245 cm^−1^ and 1180 cm^−1^) bands (**C**).

The chemical structure changes of the PHBV and the PHBV/TiO_2_ samples before and after the weathering test were investigated by FTIR. It can be seen in Figure 6, the PHBV presents the characteristic bands of the neat polymer identified in previous works [25,31,32,34,72,73,93], i.e., the observed region of 3000–2800 cm^−1^ is assigned to the asymmetric and symmetric deformations in the methylene chains (–CH_2_–), and the bands at 1472, 1448, 1425, 1338, 1335 and 1313 cm^−1^ are due to the CH_2_ and CH_3_ asymmetric and symmetric deformations, and the C–O stretching characteristic bands are seen at 1255–1245 cm^−1^ for the crystalline domains, while the amorphous phase is indicated by the 1180 cm^−1^ peak. The strong absorbance peaks at 1713 and 1720 cm^−1^ are attributed to the stretching vibrations of the crystalline C=O carbonyl groups, while the absorption bands at 1730 and 1740 cm^−1^ are assigned to the amorphous C=O stretching. The 1000–800 cm^−1^ area contains alternating spectral peaks from C–C stretching that are also sensitive to the crystallinity changes during degradation.

**Figure 7:**
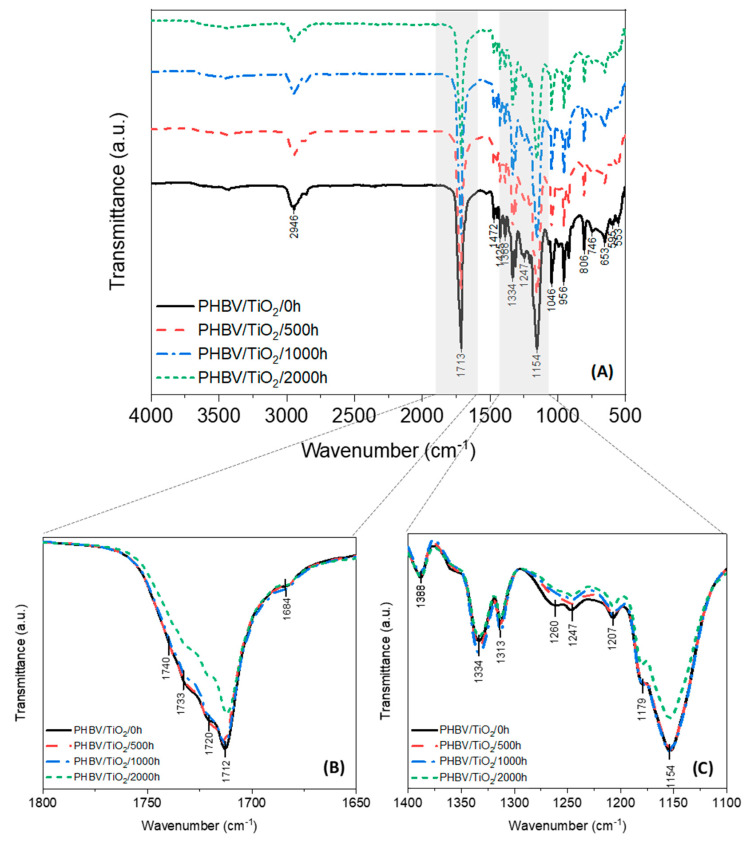
FTIR spectra of the PHBV/TiO_2_ nanocomposites before (0 h) and after (500, 1000 and 2000 h) accelerated weathering (**A**). Detailed area of: the stretching C=O vibration (**B**), the CH_3_ deformations (1338 cm^−1^), and the stretching C–O (1255−1245 cm^−1^ and 1180 cm^−1^) bands (**C**).

Accelerated weathering deteriorated the structure of the PHBV via polymer cleavage. The first stage of PHBV degradation, resulting from moisture and UV exposure, is usually the reduction of its molecular weight that occurs as a result of the chain scission of the polymer as already mentioned. Photodegradation occurred via the Norrish mechanism, especially the Norrish II type, which involves the random chain scission of ester bonds and the presence of hydroperoxide and the C=C double bond [32,34,37,88,89,90,91]. Based on the FTIR spectra of PHBV (Figure 6) and PHBV/TiO_2_ (Figure 7), it can be deduced that the chemical structure of the samples was not necessarily changed, because no vibration modes were suppressed or appeared due to degradation. However, the intensities of the C=O, C–C and C–O–C vibrations changed after degradation. The intensity of the C–O stretching of the crystalline phase observed at 1245 cm^−1^ decreased over the weathering time. In addition, a decrease in the peak at 1180 cm^−1^, corresponding to the C–O stretching of the amorphous phase and the C–C (1000–800 cm^−1^) ester bonds was also observed, such as the ester carbonyl C=O peaks assigned to both the amorphous and crystalline phases (1800–1650 cm^−1^). The detailed FTIR spectra of PHBV are shown in Figure 6.

A significantly different behavior was observed for the PHBV/TiO_2_ nanocomposites over the weathering period (Figure 7). No decrease in the intensities of the carbonyl bands was observed for the PHBV/TiO_2_ nanocomposites up to 1000 h of weathering exposure, while evidence of degradation was only observable after 2000 h. The degradation also seemed to occur simultaneous in both the amorphous and crystalline regions, as observed for the neat PHBV.

To further confirm the extent of the degradation of each phase, the carbonyl index (ratio of the intensity heights of C=O/CH_3_) was quantified before and after accelerated weathering. The absorbance ratios of 1720/1388 and 1713/1388 cm^−1^ were used to quantify the crystalline C=O of the PHBV and its nanocomposites, whereas 1730/1388 and 1740/1388 cm^−1^ were used to measure the amorphous carbonyl content [31,32,72,73]. The quantitative analysis of the carbonyl index and the ratio of crystalline/amorphous phases are presented in Table 1. It is shown that the intensity of the amorphous bands for the neat PHBV decreased over the exposure time. In studies on PHBV exposed to moisture, thermal, and UV conditions, degradation was reported to start in the amorphous phase and then to proceed into the crystalline phase [25,26,94]. The results shown in Table 1 reveal that the degradation of neat PHBV under moisture and UV exposure occurs preferentially in the amorphous phase, as evidenced by the decrease in the corresponding carbonyl index. No significant change was observed in the crystalline C=O index over the weathering time. As a result of this, an increase in the crystallinity ratio was observed for the neat PHBV.

The carbonyl content of the PHBV/TiO_2_ samples have similar values over the weathering period. Rutile TiO_2_ can be used as a UV blocker because of its great refractivity and notable chemical inertia [50,51,62]. Thus, a slight increase in the crystallinity ratio of the PHBV/TiO_2_ samples was only recorded after 2000 h of weathering and happened simultaneously in both regions, decreasing all the carbonyl indices as observed in Table 1. Some authors found that nanoclays may retard the degradation of aliphatic polyesters due to the enhanced barrier properties [26,41]. However, other authors reported a catalytic effect of clays on the hydrolytic degradation of different aliphatic polyesters, due to the high hydrophilicity of these nanoparticles [26]. These results demonstrate that TiO_2_ is not accelerating the degradation of PHBV, corroborating with results already discussed in this paper. TiO_2_ particles seem to be acting as barriers when blended with PHBV. The main reason could be the poor affinity between TiO_2_ and PHBV, which was previously demonstrated [95] and also prevent the easier permeability of water into the polymer matrix, demoting both the UV and hydrolytic degradation processes.

### 3.5. Thermal Properties

**Figure 8:**
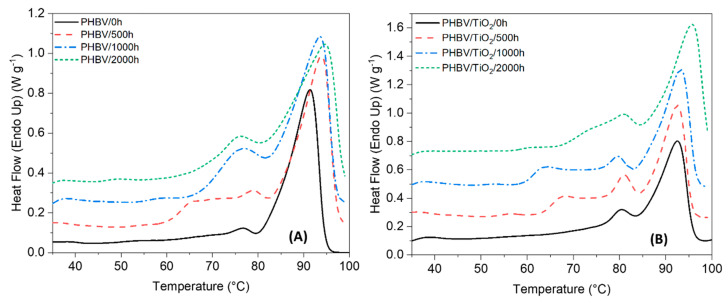
Differential scanning calorimetry (DSC) first heating curves of (**A**) the PHBV and (**B**) the PHBV/TiO_2_ nanocomposites, before and after the different periods of accelerated weathering degradation.

**Figure 9:**
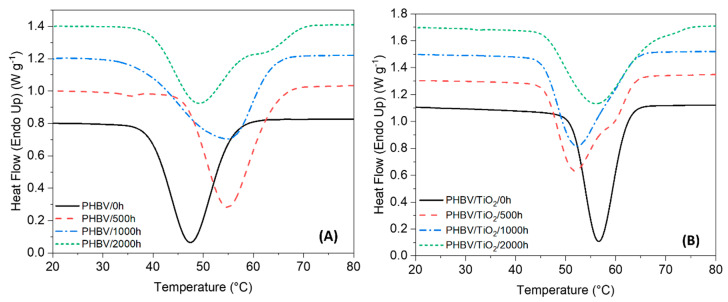
DSC cooling curves of (**A**) the PHBV and (**B**) the PHBV/TiO_2_ nanocomposites, before and after the different periods of accelerated weathering degradation.

DSC analysis was used to determine the changes in the degree of crystallinity characterized by the enthalpy and melting phenomena of PHBV and the PHBV/TiO_2_ nanocomposites after 500, 1000 and 2000 h of accelerated weathering. The DSC curves for the first heating and cooling scans are presented in Figure 8 and Figure 9, while the thermal parameters (first heating and cooling melting and crystallization enthalpies, as well as melting and crystallization temperatures, second heat glass transitions and first heat degrees of crystallinity, respectively ΔH_m_, ΔH_c_, T_m_, T_c_, T_g_ and X_c_), are summarized in Table 2.

The DSC curve of the neat PHBV shows two endothermic peaks (Figure 8). At 77 °C there is a weak melting peak, while a well resolved melting peak was observed around 92 °C. There is no single explanation for the presence of the double melting peak. The most common explanation that has been proposed include (i) the presence of more than one crystallographic form; (ii) the presence of melting, recrystallisation and re-melting; and (iii) changes in the morphology, such as lamellar thickening and crystal perfection [25,26,96].

There were no significant changes in the low-temperature melting peak of PHBV with the increase in weathering time. However, both endothermic peaks became broader and overlapped, which made it challenging to identify the temperature range of each peak after 500, 1000 or 2000 h of weathering exposure. The broadness in the melting peak is related to the broadening in the distribution of the polymer chain lengths, i.e., having both long and short chains at the same time after degradation. In addition, a small shoulder at a lower temperature (around 65 °C) was formed in PHBV/500 h, which possibly reflects a small quantity of a very low M_W_ polymer as a result of chain scission during degradation [15,58,96,97,98].

Similar behavior was observed in the DSC curves of the PHBV/TiO_2_ nanocomposites, showing a double melting peak in the first heating curve (Figure 8). The low-temperature peak is observed around 80 °C, and it is almost constant over the weathering exposure for the PHBV/TiO_2_ system. This is also observed for unloaded PHBV. According to some authors [58], the low-temperature peak is usually considered the true melting peak.

Table 2 shows that the T_m1_ of PHBV/TiO_2_/0 h was about 3 °C higher than that of PHBV/0 h, but the degrees of crystallinity were about the same. This indicates that the crystals in the case of PHBV/TiO_2_ were larger, although the degree of crystallinity did not change. This confirms that the nanoparticles acted as nucleation and growth centers for PHBV, giving rise to larger crystals around the TiO_2_ particles [95,99].

The most intense peak slightly increased from 93 to 96 °C, respectively, for PHBV/TiO_2_/0 h and PHBV/TiO_2_/2000 h. As observed for the neat PHBV, the PHBV/TiO_2_ nanocomposites also show a third melting peak which is attributed to changes in the physical structure of the polymer chains during degradation, leading to the formation of crystal populations with different perfections and lamellar thicknesses generated by the re-organization of the heterogenous crystallinity in the presence of TiO_2_ [15,25,26,55,58,96,97,98].

Significant differences between the neat PHBV and the nanocomposites are observed in the T_g_ values (Table 2). The T_g_ values did not change with increasing weathering time for the nanocomposite, while there was an increase in these values with increasing weathering time for neat PHBV. This suggests that the smaller crystallites, formed during UV-initiated degradation, restrained the chain movement so that higher temperatures were needed for the amorphous chains to become mobile [70,71].

The neat PHBV and its composites revealed some changes in crystallinity over the accelerated weathering tests. Up to 1000 h of exposure, an increase in the crystallinity of PHBV was observed, but a decrease was observed after 2000 h, while PHBV/TiO_2_ just showed a slight increase after 500 h of weathering exposure. This behavior in the crystallinity index of the samples suggests that the degradation of neat PHBV occurred preferentially in the amorphous phase, leading to chain rearrangement and re-crystallization [25,26,94], as was already suggested by the other results. A decrease in the crystallinity index after a long time of degradation exposure was observed, because the non-amorphous phase was also attacked and destroyed. The crystallinity of the PHBV/TiO_2_ samples slightly increased after 500 h of exposure, and the nucleating effect of TiO_2_ could be the reason for this, since the amorphous phase seems not to be damaged over the exposure time. Since the accelerated weathering test was run at 50 °C (T > T_g_), the reorganization of some polymeric chains was possible. After this time, there was a small continuous decrease in crystallinity, much less than in the case of neat PHBV. The presence of TiO_2_ not only restricted the polymer chain mobility, but also protected it from moisture and UV degradation. When the barrier effect of the TiO_2_ was not effective any more, the degradation was equally pronounced in the amorphous and crystalline regions.

Investigating the cooling curves of the PHBV and the PHBV/TiO_2_ nanocomposites, it can be seen that the neat PHBV shows an intense crystallization peak at 48 °C. The nanocomposite PHBV/TiO_2_/0 h presents an equally well defined but less broad crystallization peak at a higher temperature (57 °C). The higher crystallization temperature confirms the nucleating effect of the nanoparticles in the PHBV. This crystallization peak is broader and less well defined for the weathered samples, and the crystallization occurred at higher temperatures after degradation. The presence of polymer chains characterized by different molecular weights and different lengths results in a wider melting or crystallization temperature range [98]. When crystallization occurs over a broader temperature range, it means that the degradation process reduced the rate of crystallization of PHBV, affecting the morphologies of its crystals. The crystallization degree is higher for the weathered samples, which is the result of the easier re-organization of the shorter polymer chains after chain scission during degradation, as previously explained [15,25,26,55,58,94,96,97,98].

The presence of the TiO_2_ in the PHBV showed fluctuating values for the temperature and rate of crystallization over the exposure time, because of the heterogeneous bulk dispersion of TiO_2_ and its nucleation effect during the crystallization and degradation process. Higher T_m1_ and T_m2_ were recorded for the TiO_2_-containing samples, indicating that more crystal perfection with higher lamellar thickness was achieved in the presence of the titania.

From this section, we observe that the crystallinity of the nanocomposites was always lower than that of the neat polymer. The presence of TiO_2_ not only provided UV protection for the PHBV, but also restricted the mobility of the polymer chains and/or acted as a nucleating agent during the crystallization and degradation process. An analogous nucleating role on PHBV crystallization had previously been reported for other nanofillers like multi-walled carbon nanotubes (MWCNTs) [100], SiO_2_ [101]**,** and ZnO [70].

### 3.6. Thermogravimetric Analysis (TGA)

Table 3 shows the decomposition temperatures at 5 and 50% of mass loss for the neat PHBV and the PHBV/TiO_2_ nanocomposite after the different periods of artificial weathering. Only one degradation step was observed for the neat polymer and the nanocomposite. A single-step degradation process was a good indicator that the degradation was homogeneous throughout the thickness of the specimen [102].

Neat PHBV has an initial decomposition temperature around 300 °C, and a significant mass loss is observed between 300 and 450 °C, which corresponds to the decomposition of the PHBV with a maximum rate at 407 °C. This indicates a nonradical random chain scission mechanism involving a six-membered ring ester decomposition process [27]. The final degradation products of PHBV are CO_2_ and H_2_O [45]. After this, only a gradual mass loss was observed until a constant mass was reached. The degradation temperatures corresponding to a 5 and 50% mass loss of PHBV were found to be 348 and 398 °C. Similar TGA curves were observed for the PHBV before and after weathering. However, after 2000 h of weathering exposure, the degradation of the neat PHBV started at a significantly lower temperature. The degradation temperatures at 5 and 50% of mass loss, that are presented in Table 3, show a decrease over the weathering time from 348 to 237 °C and 398 to 379 °C, respectively.

In the case of the nanocomposite, the decomposition occurred over a similar temperature range than that of the neat PHBV, between 300 and 450 °C. In all the samples, similar TGA and dTGA curves were observed for the PHBV/TiO_2_ nanocomposite before and after 500 and 1000 h of weathering. The degradation temperatures at 5 and 50% of mass loss, which are presented in Table 3, show a slight decrease over the weathering time from 348 to 345 °C, and from 399 to 395 °C, respectively, for PHBV/TiO_2_/0 h and PHBV/TiO_2_/2000 h.

It should be noted that the addition of TiO_2_ to PHBV did not significantly change the thermal stability of the PHBV in the nanocomposites up to 1000 h of UV exposure. Some authors report that TiO_2_ nanoparticles containing moisture can take part in the degradation process at elevated temperatures, which accelerated the thermal and hydrolysis degradation that co-occur [43]. Others defend a faster degradation rate for neat polymers than for the nanocomposites, and attribute it to the TiO_2_ particles acting as heat barriers in the early stages of thermal decomposition, which improves the thermal stability of the nanocomposite [25,103,104]. This behavior was not observed in our study for samples up to 1000 h of UV exposure, and the TGA and dTGA curves revealed that the PHBV and PHBV/TiO_2_ had similar characteristic degradation temperatures over this degradation period.

The nanocomposite shows a higher thermal stability than the neat PHBV for the 2000 h UV exposed samples. The initial decomposition temperature shifted to lower temperatures when TiO_2_ was not present, which indicates a decrease in the polymer stability after this weathering time in the neat PHBV samples. In accordance with previous results, the weathering degradation process was delayed in the presence of the TiO_2_ nanoparticles. The constant mass remaining at the end of each TGA experiment corresponds to the amount of inorganic material, i.e., the TiO_2_, which is very close to the theoretical amount of particles in the nanocomposite.

### 3.7. Melt Flow Index (MFI)

MFI measurement is a common technique to study the flow behavior of polymers. MFI does not take into account the variation in the shear rate, but it is a simple test that can potentially confirm conclusions from other results.

Table 4 shows the MFI values of neat PHBV and the PHBV/TiO_2_ nanocomposite before and after accelerated weathering. In the case of neat PHBV there is a strong increase in MFI from 6 to 32 g/10 min., which did not change significantly for the 1000 h sample. This indicates a significant decrease in viscosity, which supports the previous conclusion of degradative chain scission. Since polymer viscosity depends on chain length, the much lower viscosity confirms the formation of much shorter chains during the weathering degradation of the neat polymer. No value could be determined for the 2000 h exposed sample, because the sample was so degraded that it flowed without the application of load.

The unexposed nanocomposite has a similar MFI than the unexposed PHBV. For the nanocomposite, the MFI gradually increased up to the 1000 h exposure (Table 4), indicating a limited decrease in viscosity accompanying a slight degradation, after which (for the 2000 h sample) it more than doubled to a value comparable to that of the 500 h PHBV sample. This confirms our previous conclusions that the TiO_2_ protected the PHBV from weathering degradation for up to 1000 h exposure, but then it also degraded significantly over the next 1000 h.

### 3.8. Mechanical Properties

#### 3.8.1. Bulk Properties

**Figure 10:**
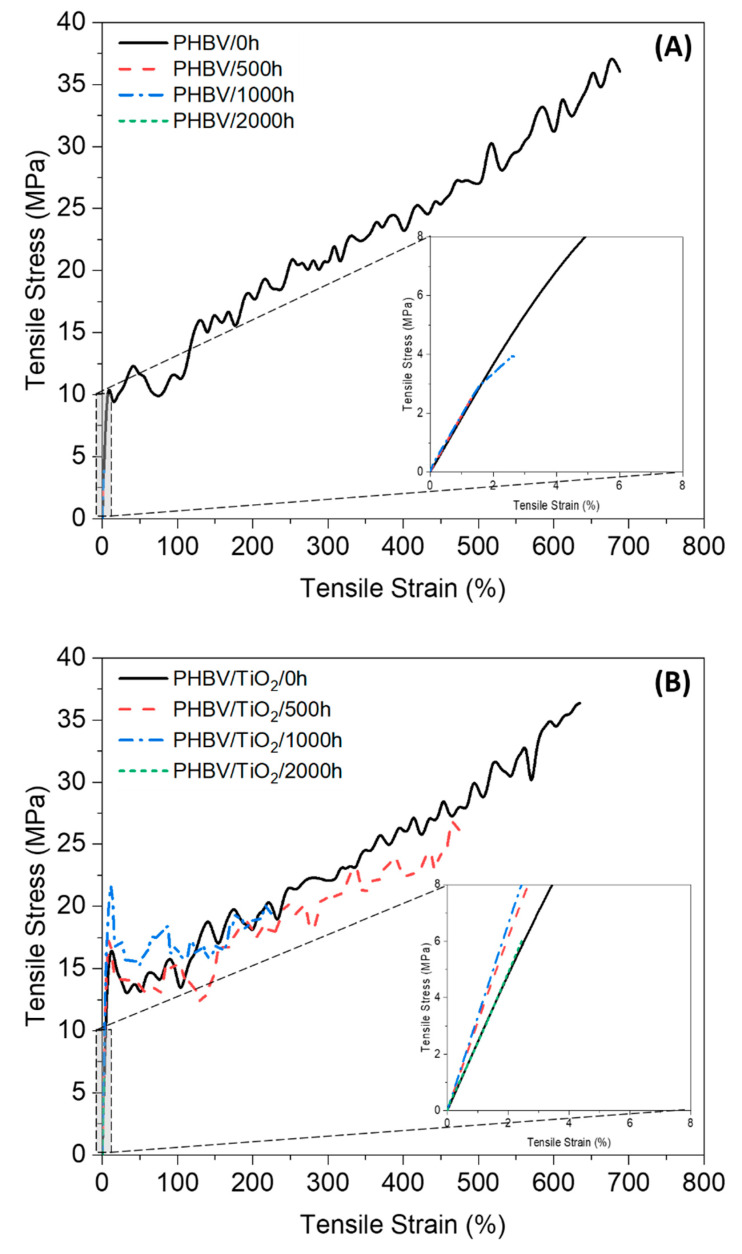
Representative tensile stress–strain curves of (**A**) the neat PHBV and (**B**) PHBV/TiO_2_ samples after the different periods of accelerated weathering time.

Tensile tests were carried out on the PHBV samples and the PHBV/TiO_2_ composites after the different periods of accelerated weathering. Figure 10 presents an example of each stress–strain curve of all the studied samples, while the tensile properties are summarized in Table 5.

Before weathering, the PHBV samples had a Young’s modulus (E), strength at break (σ_break_) and elongation at break (ε_break_) values of 182 ± 6 MPa, 34.2 ± 1.7 MPa and 730 ± 40%, respectively. For the 500 h exposed samples, it was observed that the E remained fairly constant, while the ε_break_ and σ_break_ decreased to 1.1 ± 0.3% and 2.0 ± 0.6 MPa, respectively. Mechanical properties generally decrease with increasing weathering exposure time, as observed by other authors [22,34,94], because physical and chemical changes occur in the polymer structure, as already discussed. The mechanical properties of neat PHBV decrease over the weathering time, mostly due to a decrease in M_W_ [22,26,34,94]. These results corroborate with the changes in T_g_, where the temperature increased over exposure time, indicating shorter chains in the amorphous phase. In addition, the crystallinity indices increased as observed in the DSC and XRD data, which was enough reason for E to remain constant, but for ε_break_ and σ_break_ to decrease. Some authors also suggested a possible crosslinking effect during degradation, which enhanced the stiffness of the material [26,34]. When the exposure time of the neat PHBV was 1000 h, the σ_break_ and ε_break_ values remained approximately the same, because they were already very low and could not decrease much more. The Young’s modulus decreased fairly significantly for this sample, confirming the decrease in molecular weight because of the UV-initiated degradation. The PHBV/2000 h samples were not tested because they were significantly cracked and brittle.

The unexposed PHBV/TiO_2_ showed a significantly higher value for E (238 ± 8 MPa), and similar values for σ_break_ (36.3 ± 0.2 MPa) and ε_break_ (672 ± 34%) than the neat PHBV. In addition, a significantly different profile of the weathering degradation was observed for the PHBV/TiO_2_ nanocomposites. The σ_break_ and ε_break_ gradually decreased with exposure time, while the E slightly increased, in contrast with the neat polymer, where these values significantly decreased with increasing exposure time. These differences in the mechanical properties between the TiO_2_-loaded and -unloaded PHBV after the accelerated weathering could have been caused by various factors. First of all, the addition of fillers generally enhances the mechanical properties of polymer composites [105]. Secondly, the TiO_2_ nanoparticles presented a nucleation effect, leading to the formation of thinner crystals during both the preparation and degradation of the samples [106]. Lastly, the TiO_2_ particles showed a low affinity for PHBV [95,99], acting as a protection barrier during the weathering attack. This behavior retarded the degradation process under the photo and moisture exposure, shifting the degradation process to longer periods of time.

Another interesting result was the slightly higher E observed for PHBV/TiO_2_/1000 h compared to the samples exposed for shorter periods. Despite the decrease in the degree of crystallinity after this time, the presence of the nanofiller seemed to have maintained the mobility constraint of the PHBV molecular chains during the weathering exposure.

A sharp drop was observed for the PHBV/TiO_2_/2000 h, showing that both the amorphous and crystalline phases were available for degradation, changing their physical and chemical characteristics as previously reported, leading to poor mechanical properties. This is an important point for some kind of polymer application when biodegradability is desired, but it is not acceptable during the shelf life of the product but only wanted at the end of use [107].

#### 3.8.2. Surface Properties

**Figure 11:**
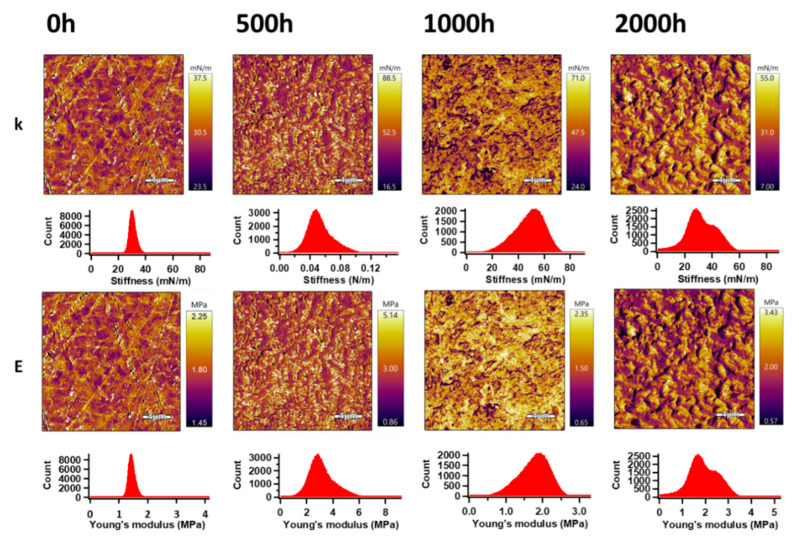
**A**mplitude modulation–frequency modulation (AM–FM) images (upper) and histograms (lower) of the PHBV samples, before and after the accelerated weathering; **k** and **E** represent the stiffness and Young’s modulus, respectively.

**Figure 12:**
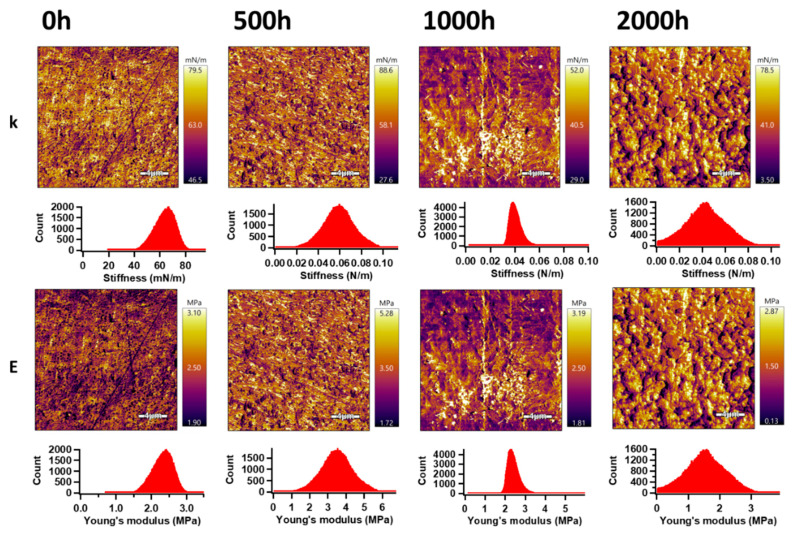
AM–FM images and histograms of the PHBV/TiO_2_ samples before and after the accelerated weathering; **k** and **E** represent the stiffness and Young’s modulus, respectively.

The AM–FM mode (tool of AFM) was used for the analysis of the mechanical properties on the surface of the PHBV and the PHBV/TiO_2_ nanocomposites. Using this mode, the information about stiffness (k) and Young’s modulus distribution on the entire surface area of the analyzed samples, before and after the accelerated weathering exposure, were obtained and are shown in Figure 11 and Figure 12. Gaussian fitting [108] was applied for an evaluation of the k and E mean values (obtained by different approaches) from the peaks in the respective histograms (Table 6). For PHBV, these values were 0.030 N m^−1^ and 1.78 MPa, respectively. After 500 h of accelerated weathering, the values of k and E increased to 0.049 N m^−1^ and 2.90 MPa, respectively. Increasing weathering exposure time up to 2000 h was responsible for the decrease in the surface mechanical properties, while k and E achieved values of 0.031 N m^−1^ and 1.86 MPa. These results are in accordance with the increased crystallinity observed from the DSC results for up to 500 h of weathering time, after which the degradation effect became more dominant in the surface area.

As opposed to the heterogeneous bulk dispersion of TiO_2_ [95,99], the TiO_2_ in the surface of the PHBV/TiO_2_ samples seemed to have been well distributed and improved their mechanical properties. The values of k and E increased up to 0.057 N m^−1^ and 2.33 MPa, respectively, for the unweathered PHBV/TiO_2_. Longer exposure times up to 1000 h showed a similarly affected E of the PHBV/TiO_2_ samples as in the case of the neat PHBV samples, but achieving slightly higher values because TiO_2_ acted as a degradation retarder. However, 2000 h of accelerated weathering of PHBV/TiO_2_ showed a remarkably decrease in E to a value of 1.53 MPa, which is slightly less than in the case of neat PHBV. The peak width values indicate the presence of much broader peaks (consisting of two overlapped peaks), confirming our previous DSC observation of the simultaneous presence of the crystalline and amorphous phases. The difference in the degree of softness of these two phases would definitely affect the mechanical surface properties. These results confirm the good stability of the mechanical properties of the PHBV/TiO_2_ nanocomposite in the surface area during at least the first 1000 h of accelerated weathering, because TiO_2_ retarded the hydrolysis and UV degradation processes.

## 4. Conclusions

The purpose of this work was to characterize the PHBV and the PHBV loaded with 3 wt.% of TiO_2_ nanoparticles, before and after the accelerated weathering (UV and moisture conditions) over periods of 500, 1000 and 2000 h. Accelerated weathering exposure had a significant effect on the general properties of the PHBV samples as a result of degradation. The PHBV was initially ductile and presented a smooth surface, which changed during the weathering exposure time, showing the development of cracks and holes, while the PHBV/TiO_2_ nanocomposites was less susceptible to degradation.

The XRD, DSC and FTIR analysis showed an overall increase in crystallinity after the accelerated weathering for the neat PHBV samples. The intensities of the XRD peaks were lower for the nanocomposites and only small changes in the carbonyl bands after 2000 h of weathering exposure were observed for the PHBV/TiO_2_ samples, suggesting a more extensive degradation of PHBV in the absence of TiO_2_, which is in line with the AFM and SEM observations.

The FTIR and DSC results confirmed that the first stage of the weathering degradation of PHBV was the cleavage of amorphous polymer chains. The smaller carbon chain length justified the hydrophilicity of the neat PHBV samples after 500 h of weathering, although the higher mobility led to chain re-organization and also promoted the crystallinity of the samples. TiO_2_ prevented hydrolysis and UV attack, which could be observed in the unchanged T_g_ values for the nanocomposites, while neat PHBV presented an increase in this temperature over the exposure time.

One thermal degradation step was observed in the TGA for both the neat PHBV and the PHBV/TiO_2_ nanocomposite. The temperature values of the thermal degradation of the neat PHBV tended to be similar up to 1000 h of weathering, but after 2000 h, this temperature shifted to lower values compared to those of the PHBV/TiO_2_ nanocomposites.

The surface and bulk mechanical properties of the PHBV and the PHBV/TiO_2_ were also affected by accelerated weathering as a result of degradation processes. A significant decrease in these properties was observed for the neat PHBV samples with increasing weathering time, contrary to the gradual changes of samples loaded with TiO_2_.

Only after 2000 h of weathering exposure, the nanocomposites showed significant changes in their physical, chemical and mechanical properties. However, these were still less prominent than those observed in the neat PHBV samples. The addition of TiO_2_ enhanced the mechanical properties of the nanocomposites, presented a nucleation effect during both the preparation and degradation of the samples, and acted as a protection barrier during the weathering attack which retarded the degradation process under photo and moisture exposure, shifting the degradation process to longer periods of time.

## Figures and Tables

**Table 1:** Carbonyl index and crystalline/amorphous ratios for the PHBV and the PHBV/TiO_2_ nanocomposites, both before and after weathering.

	Wavenumbers (cm^−1^)							
	Carbonyl Index				Ratio of crystalline/amorphous			
	C=O crystalline		C=O amorphous					
	1720/1388	1713/1388	1730/1388	1740/1388	1720/1740	1713/1740	1720/1730	1713/1730
PHBV/0 h	2.7	3.1	2.3	1.7	1.6	1.9	1.2	1.4
PHBV/500 h	2.7	3.2	2.0	1.5	1.8	2.1	1.3	1.6
PHBV/1000 h	2.7	3.3	2.0	1.4	1.9	2.3	1.4	1.7
PHBV/2000 h	2.5	3.2	1.6	1.1	2.2	2.8	1.5	2.0
PHBV/TiO_2_/0 h	2.8	3.3	2.2	1.7	1.7	2.0	1.3	1.5
PHBV/TiO_2_/500 h	2.9	3.2	2.2	1.7	1.7	1.9	1.3	1.4
PHBV/TiO_2_/1000 h	2.8	3.3	2.1	1.6	1.7	2.0	1.3	1.5
PHBV/TiO_2_/2000 h	2.5	3.2	1.9	1.5	1.7	2.2	1.3	1.7

**Table 2:** DSC data obtained for the neat PHBV and the PHBV/TiO_2_ nanocomposites after accelerated weathering degradation.

	1st Heating					Cooling		2nd Heating
	T_m1_ (°C)	T_m2_ (°C)	T_m3_ (°C)	ΔH_m_ (J g^−1^)	X_c_ (%)	T_C_ (°C)	ΔH_C_ (J g^−1^)	T_g_ (°C)
PHBV/0 h	76.6	91.5		58.8	53.7	47.5	47.0	−47.3
PHBV/500 h	78.8	94.1	65.3	61.8	56.4	54.9	47.0	−43.4
PHBV/1000 h	76.5	93.7	58.8	65.0	59.4	55.0	52.5	−41.3
PHBV/2000 h	75.9	94.8		57.3	52.3	49.5 / 62.4	44.5	−40.7
PHBV/TiO_2_/0 h	80.2	92.6		55.2	52.0	56.7	39.7	−43.3
PHBV/TiO_2_/500 h	81.1	92.6	56.3	57.7	54.3	51.9 / 59.5	42.9	−43.6
PHBV/TiO_2_/1000 h	79.8	93.3	63.8	53.7	50.6	52.3	42.1	−43.5
PHBV/TiO_2_/2000 h	81.1	95.9		49.6	46.7	56.3	42.3	−43.0

**Table 3:** Characteristic degradation temperatures for the PHBV and the PHBV/TiO_2_ samples obtained from the TGA and derivative TGA (dTGA) curves (not shown).

Sample	T_5%_ (°C)	T_50%_ (°C)	T_max_ (°C)
PHBV/0 h	348	398	407
PHBV/500 h	355	399	407
PHBV/1000 h	350	400	409
PHBV/2000 h	237	379	394
PHBV/TiO_2_/0 h	348	399	407
PHBV/TiO_2_/500 h	352	397	404
PHBV/TiO_2_/1000 h	343	393	398
PHBV/TiO_2_/2000 h	345	395	403

**Table 4:** Changes in the MFI of the PHBV and the PHBV/TiO_2_ samples as a function of accelerated weathering time.

Sample	MFI (g/10 min.)
PHBV/0 h	5.6 ± 0.1
PHBV/500 h	31.9 ± 3.0
PHBV/1000 h	35.1 ± 4.1
PHBV/2000 h	−
PHBV/TiO_2_/0 h	4.2 ± 0.5
PHBV/TiO_2_/500 h	8.1 ± 0.1
PHBV/TiO_2_/1000 h	16.1 ± 1.5
PHBV/TiO_2_/2000 h	34.1 ± 4.8

**Table 5:** Mechanical properties of the neat PHBV and the PHBV/TiO_2_ after the different periods of accelerated weathering.

Sample	σ _break_ (MPa)	ε _break_ (%)	E (MPa)
PHBV/0 h	34.2 ± 1.7	730 ± 40	181 ± 6
PHBV/500 h	2.0 ± 0.6	1.1 ± 0.3	168 ± 39
PHBV/1000 h	1.7 ± 1.6	3.6 ± 0.5	79.2 ± 65.9
PHBV/2000 h	−	−	−
PHBV/TiO_2_/0 h	36.3 ± 0.2	672 ± 34	238 ± 8
PHBV/ TiO_2_/500 h	27.2 ± 1.4	520 ± 44	296 ± 22
PHBV/ TiO_2_/1000 h	21.5 ± 1.9	241 ± 26	366 ± 61
PHBV/ TiO_2_/2000 h	7.8 ± 0.5	2.6 ± 0.1	177 ± 7

**Table 6:** Surface mechanical properties of the PHBV and the PHBV/TiO_2_ nanocomposites collected from the AFM analyses.

Sample	Stiffness (N m^−1^)		Young’s Modulus (MPa)	
	Mean	Width	Mean	Width
PHBV/0 h	0.030	0.002	1.78	0.14
PHBV/500 h	0.049	0.014	2.90	0.83
PHBV /1000 h	0.050	0.011	1.80	0.41
PHBV /2000 h	0.031	0.011	1.86	0.66
PHBV /TiO_2_/0 h	0.064	0.008	2.33	0.30
PHBV /TiO_2_/500 h	0.058	0.014	3.46	0.84
PHBV /TiO_2_/1000 h	0.039	0.004	2.31	0.25
PHBV /TiO_2_/2000 h	0.042	0.017	1.53	0.65
